# Validation of the Arabic version of the resilience scale for adolescents (READ)

**DOI:** 10.1186/s12888-023-05219-4

**Published:** 2023-10-02

**Authors:** Roni Chaaya, Sahar Obeid, Alvaro Postigo, Dina Dagher, Rabih Hallit, Diana Malaeb, Mariam Dabbous, Fouad Sakr, Feten Fekih-Romdhane, Souheil Hallit

**Affiliations:** 1 Social and Education Sciences Department, School of Arts and Sciences, Lebanese American University, Jbeil, Lebanon; 2 Department of Psychology, University of Oviedo, Oviedo, Spain; 3 School of Medicine and Medical Sciences, Holy Spirit University of Kaslik, P.O. Box 446, Jounieh, Lebanon; 4 Department of Infectious Disease, Bellevue Medical Center, Mansourieh, Lebanon; 5 Department of Infectious Disease, Notre Dame des Secours, University Hospital Center, Postal Code 3, Byblos, Lebanon; 6 College of Pharmacy, Gulf Medical University, Ajman, United Arab Emirates; 7 School of Pharmacy, Lebanese International University, Beirut, Lebanon; 8 The Tunisian Center of Early Intervention in Psychosis, Department of psychiatry “Ibn Omrane”, Razi Hospital, Manouba, 2010 Tunisia; 9 Faculty of Medicine of Tunis, Tunis El Manar University, Tunis, Tunisia; 10 Psychology Department, College of Humanities, Effat University, Jeddah, 21478 Saudi Arabia; 11 Applied Science Research Center, Applied Science Private University, Amman, Jordan; 12 Research Department, Psychiatric Hospital of the Cross, Jal Eddib, Lebanon

**Keywords:** Resilience, READ, Adolescents, Arabic, Psychometric properties

## Abstract

**Introduction:**

Adolescents react differently to challenging negative life events. Resilience, a dynamic characteristic of individuals, was studied to be a protective factor against such events. In order to study the resilience among Arabic-speaking adolescent populations, age-appropriate measures that are fully apprehended by younger respondents are needed. In this context, the present study aimed to examine the psychometric properties of an Arabic translation of the Resilience Scale for Adolescents (READ) in a community sample of native Arabic–speaking adolescents aged 13–18 years.

**Methods:**

A sample of 546 community Arabic-speaking adolescents from Lebanon was recruited (n = 328 females, with a mean age of 15.76 ± 1.65 years). Through an online questionnaire, participants were requested to complete the READ, Depression, Anxiety and Stress Scale (DASS-8) and the 13-item Children’s Impact of Event Scale (CRIES-13).

**Results:**

Following the exploratory and confirmatory factor analysis (EFA-to-CFA) strategy, a unidimensional model of the Arabic version of the READ was met after 10 items were removed from the scale, and showed strong internal consistency (Cronbach’s alpha of 0.943). Additionally, the one-factor solution of the Arabic version of the READ was identical across male and female adolescents at the three levels of invariance (Configural, Metric and Scalar). Finally, higher resilience scores were significantly correlated with lower levels of psychopathology, namely depression, anxiety, stress and PTSD, thus attesting to the concurrent validity of the Arabic READ.

**Conclusion:**

Findings lend support to the psychometric reliability and validity of the Arabic version of the READ, and therefore its suitability for use among Arabic-speaking adolescents. The availability of this tool facilitates the implementation of interventions that foster resilience, especially in adolescents who have faced a number of negative life events.

## Introduction

The transition from childhood to adolescence involves concurrent physical and psychological transformations that influence several aspects of brain development, social cognition, and peer relationships. These transformations have also proven to be closely related to risk for psychological distress [1]. For instance, depression has been witnessed to experience sharp surges during puberty [2]. with Arab adolescents being not an exception to this. Arab countries have one of the youngest age structures worldwide, with around 60% of the population being under 25 years old [3]. Mental health issues are highly prevalent in Arab adolescents (e.g., 32.4% of 960 Saudi adolescents had moderate to severe depression [4], 34% of 2,349 Jordanian adolescents reported moderate to severe depression [5] and 28% of 968 Emirati adolescents had anxiety disorder [6]) and were found to contribute to nearly 25% of Arab adolescents’ disability [7]. Mental health burden was shown to be particularly alarming in Arab adolescents who live in areas of armed conflict [8]. Indeed, early life stress and potentially traumatic events represent one of the major predictors of mental health problems in adolescents [9]. However, not all adolescents who experience such challenging negative life events will witness any psychological distress later in their lives [10].

Noting that different people react differently to life events and traumatic occurrences [11], researchers sought to understand individual qualities and characteristics of people capable of thriving in light of adversities faced in life [12]. One of these identified qualities is ‘resilience’, which highlights one’s ability to demonstrate positive adaptation in spite of encountering significant life adversities [13]. Resilience is a concept that has been gaining a lot of traction lately and is being frequently employed to describe how individuals and groups of people cope and deal with traumatic and distressing situations [14]. Even though there is not a common agreement as to what the best definition of resilience is, there is a general consensus on three overarching protective characteristics of resilience which make it a multidimensional construct. The first characteristic relates to positive personal attributes and characteristics of an individual. In elaboration, these consist of being skilled socially, showing healthy emotions, and empathy towards others. Secondly, having a family that is supportive is another protective characteristic of resilience which refers to having an emotionally supportive family that provides warmth and facilitates the development of a positive attachment for children. The third and final characteristic is having a social environment, aside from the family, that is characterized by being supportive. One’s school, neighborhood and friends constitute this social environment [15, 16].

The growing interest in resilience has been motivated by its role as a protective factor in preventing and mitigating the risk of mental health issues, particularly in the face of life stressors [16]. Resilience is not a fixed, inherent trait to individuals; however, it is a dynamic individualistic resource that evolves over the course of life. Moreover, being resilient is not synonymous to being immune to stress and rather signifies one’s capacity to effectively cope with adverse situations through the use of protective resources within oneself or in the surrounding environment [16]. In adolescence, resilience was found to be inversely proportional to trauma symptoms [17]. Moreover, interventions that focused on fostering resilience in adolescents in school, such as the FRIENDS program [18], have been proven to reduce symptoms of anxiety and depression. These results were maintained even following a one-year follow-up assessment [19]. A different study, however, found that these alleviations in the psychopathological symptoms were maintained even after 3 years from the implementation in Grade 6 students but were lost in Grade 9 students after 1 year [20]. Therefore, the authors of the study suggest that interventions that aim to reduce psychopathological symptoms and foster resilience should be conducted as early as possible, given that the results are more likely to be maintained when the intervention is implemented with younger students [21].

Although there exists a number of measures of resilience (e.g., the Resilience Scale [22], the Brief Resilience Scale [23], the Connor–Davidson Resilience Scale [24], and the Child and Youth Resilience Measure [25]), there is no general consensus on which scale is preferable over the others. A meta-analysis by Windle et al. [26] indicated that there is “no current ‘gold standard’ amongst 15 measures of resilience” to be used in children and adolescents. Thus, researchers find it difficult to select one resilience measure that can be used with all populations and settings, sometimes making unsuitable choices in their selection [26]. Moreover, the wide variety of instruments that measure resilience hinders the researchers’ abilities to compare results from different studies, especially since not all these instruments cover the aforementioned three overarching protective characteristics of resilience [16].

Among the measures specifically developed for self-report by adolescents, the Resilience Scale for Adolescents (READ) [15] is one of the most widely used. The READ is the only instrument that includes the three overarching protective factors of resilience, alongside the social dimensions of resilience-related protective factors [15]. It was developed as an adaptation of the 33-item Resilience Scale for Adults (RSA) [27], which demonstrated an excellent reliability (Cronbach’s alpha of 0.93) and was found to have a five-factor structure through an exploratory principal component analysis. To adapt the RSA for use in adolescents, items less relevant for this age group were deleted. As a result, the READ consists of 28 items, and demonstrated a matching five-factor model in a large sample of Norwegian adolescents, namely (1) personal competence, (2) social competence, (3) structured style, (4) family cohesion, and (5) social resources [15]. However, the factorial validity of the READ has led to inconsistent results. A cross-country validation showed that the five-factor model was similar in adolescents aged 10–15 years from four European countries (Spain, Iceland, Italy, Poland) [28], whereas other studies showed problematic items loading, and were able to retain five-factor solution only after removing a number of items (e.g., 26 items [29], 23 items [30], and 20 items [16]). The factors of the READ have been proven to have a Cronbach’s alpha ranging from 0.69 for the ‘structured style’ factor till 0.89 for the ’family cohesion’ factor [16, 30]. The reliability coefficient of total items of the READ tested on a Norwegian sample of adolescents was calculated to be α = 0.94 [16], while that calculated in a sample of Irish adolescents was α = 0.88 [31]. On the other hand, the Italian version was found to have an α = 0.91 [32]. As for the scale’s validity, various studies have supported its convergent validity. For instance, Hjemdal et al. (2010) found that, as the scores on the READ increased, lower scores of depression and anxiety ensued [33]. Similar results were also found between the READ scores, stress and obsessive-compulsive symptoms in a validation among 7033 Norwegian youth aged 18–20 years [30]. Furthermore, while negative life events, like the divorce of one’s parents or the death of a loved one, are associated with lower scores on the READ scale, one’s engagement in hobbies, such as sports, correlates with higher READ scores [34]. Overall, the psychometric properties of the READ have been verified in various countries and languages, including Italian [32], German [35], Spanish [29, 36], Icelandic, and Polish [28]. However, no Arabic version has been made available to date.

### The present study

The goal of the current study was to explore the psychometric properties of the READ scale, with this being an essential step for a variety of reasons. The READ was chosen because it is the only measure to comply with the resilience theoretical framework, incorporating the three salient domains recognized as mutually involved in resilience (i.e., individual, family, and external environment factors). The READ has also proven its measurement invariance across Western cultures and countries [28]. However, it is crucial to test its psychometric properties in settings and contexts other than Western European [16], in order to confirm its originally proposed factor model, and determine whether resilience has the same meaning across various cultures and populations. Moreover, Arab countries have one of the most youthful age structures in the world (around 60% of the population are under 25 years old) [37]. For instance, about 28% of Lebanon’s total population were aged 0 to 14 years in 2021 [38]. Children and adolescents in Lebanon and the broader Arab region are raised in an environment characterized by ongoing instability, violence and political upheavals [39], making resilience a major priority for practitioners and researchers working in Arab settings. Surprisingly however, and until very recently, a little amount of empirical research has been conducted on resilience among Arab adolescents [40]. Additionally, the concept of resilience has mainly been measured in westernized, high-income countries, whereas culture has an impact on the development of resilience. Given the mixed results regarding the factor structure of the READ in previous psychometric evaluations [41], the examination of its cross-cultural applicability is relevant, notably within the specific Arab context, where family is viewed as the core social organization and serves as a primary source of support [42]. Additionally, the adaptation and validation of an Arabic version of the READ may help inform the highly required implementation of a culturally tailored and sensitive resilience framework [41], and may thus serve the specific clinical and research needs of local populations. Therefore, this study aimed to examine the psychometric properties of an Arabic translation of the READ in a community sample of Arabic–speaking adolescents aged 13–18 years from Lebanon. Using the EFA-to-CFA strategy, the five-factor model of the READ is expected to ensue from the results generated from this study. Moreover, good reliability and invariance across gender groups are expected to be found. As for the validity of the Arabic version of the READ, it is expected that convergent validity will be supported through associations with measures of psychological distress and post-traumatic stress disorder (PTSD).

## Methods

### Participants

For this study, 546 adolescents from the general population of Lebanon who were aged 13–18 years were gathered from all across the Lebanese governorates (male n = 218, female n = 328), with a mean age of 15.76 years (SD = 1.65).

### Instruments

#### Resilience scale for adolescents (READ) – arabic version

The Arabic version of the READ is composed of 28, self-reported items. Adolescents are requested to assess their personal and social resilience resources based on their experiences over the past month. It consists of a Likert scale that ranges from 1 = totally agree to 5 = totally disagree, with stronger resilience being indicated by lower scores [16]. As previously mentioned, a five-factor structure was supported for the READ [15] with a total Cronbach’s alpha ranging from 0.88 to 0.94. The first factor, consisting of 8 items, is (1) *personal competence* which highlights having confidence in one’s abilities and judgments, possessing self-efficacy, and maintaining realistic expectations, with items such as “I feel competent”. The second factor, consisting of 5 items, is (2) *social competence* which encompasses qualities such as social warmth and flexibility, making friends, and using humor in a positive manner, with items such as “I easily find new friends”. The third factor, (3) *structured style*, has four items that evaluate one’s inclination to having routines and following them, being organized, and prioritizing having clear goals, with items such as “I am good at organizing my time”. The fourth item, (4) *family cohesion*, has 6 items that relate to the extent to which values, mutual appreciation and support are shared within a family, with items such as “I feel comfortable with my family”. The final factor, (5) *social resources*, consists of 5 items and demonstrates the availability of social support from friends and others beside family members, with items such as “My friends always stick together” [16]. The English version of READ was translated to Arabic by a Lebanese translator who was unrelated to the study. Following that, a Lebanese psychologist translated the Arabic version of the scale back to English. Additionally, a comparison between the Arabic and English versions of the scale was done by the research team to eliminate any inconsistencies.

#### The Depression, anxiety, and stress scale (DASS-8)

The DASS-8, a shortened version of the DASS-21, consists of eight items divided into three subscales: depression (3 items), anxiety (3 items), and stress (2 items) [43]. Responses to the items are scored on a 4-point scale, ranging from 0 (did not apply to me at all) to 3 (applied to me very much or most of the time). The overall score of the DASS-8 ranges from 0 to 24, whereas the subscale scores range from 0 to 9, 0 to 9, and 0 to 6, respectively. Higher scores equate to a higher level of symptom affirmation (McDonald’s omega (ω) = 0.87).

#### The 13-item children’s impact of event scale (CRIES-13)

This is a self-report measure used to screen for PTSD in children and adolescents (aged 8 years and older). There are two versions available. The CRIES-13 [44] includes 13 items divided into three dimensions: intrusion, avoidance and hyperarousal. Respondents rate the frequency with which they have experienced each of the items during the past week using a 4-point Likert-like scale ranging from 0 (not at all) to 5 (often). The total score indicates the severity of the posttraumatic stress response. In the present sample, probable PTSD was identified using a cut-off of 30 [45]. The Arabic version of the CRIES was used in the present study [46], and yielded a McDonald’s omega (ω) coefficient of 0.92.

### Procedure

Ethical approval for this study was obtained from the ethics committee of the School of Pharmacy at the Lebanese International University (approval code: 2023RC-009-LIUSOP). The snowball method was used to choose our sample. Using Google Forms, the questionnaire was created and shared through a convenient sampling with Lebanese adolescents during March 2023; the research team approached adolescents of their acquaintances from all Lebanese governorates (Beirut, Mount Lebanon, North, South, and Bekaa), who were later asked to forward the link to other friends and family members, preferably as diverse as possible with regard to place of habitat within the Lebanese governorates and within the same age interval required to participate in the study. The advertisement for the project was conducted through social media posts. The inclusion criteria for this study consisted of being a Lebanese citizen and resident between the age of 13 and 18 years. Informed consent was conducted electronically through the questionnaire, after which the participants were asked to fill out the aforementioned scales in the Google Form. The anonymity and confidentiality of the participants and their results was ensured alongside their voluntary participation without any remuneration. At the end of the survey, debriefing information was offered to all participants.

### Data analysis

We did not have missing values in our dataset. First, in order to study the factor structure of the scale, the sample was divided into two random subsamples: one third for Exploratory Factor Analysis (EFA) and two third for Confirmatory Factor Analysis (CFA). For the EFA (1/3, 182 participants), the Kaiser-Meyer-Olkin (KMO) and Bartlett’s statistic were used to study the adequacy of the data for the Factor Analysis. As it is a scale with 5 response alternatives, the EFA was carried out on the Pearson correlation matrix and Unweighted Least Squares (ULS) was used as the estimation method. The Measure of Sampling Adequacy (MSA) [47] was used to study the adequacy of each of the items for the EFA, whose value must be greater than 0.50. The dimensionality of the instrument was determined through the percentage of variance explained and the optimal implementation of Parallel Analysis [48] with 1,000 random correlation matrices. In addition, the Unidimensional Congruence (UniCo), Explained Common Variance (ECV), and Mean of Item REsidual Absolute Loadings (MIREAL) indices were employed to study the fit of the data to a single dimension. The following values support treating the data as essentially unidimensional: UniCo > 0.95; ECV > 0.85; MIREAL < 0.30 [49]. Factor loadings were taken into account, the criterion being that they were greater than 0.40. The Expected Residual correlation direct Change index (EREC) [50] was also used to assess the residual correlation between a pair of items once the influence of the common factors has been partialled out. Comparative Fit Index (CFI) > 0.95 and standardized root mean square residual (SRMR) < 0.06 were used to assess the good fit of the models [51]. In addition, the Pearson correlation matrix between the facets of which the READ is composed was studied.

On the other hand, with the second subsample (2/3, 364 participants), the internal structure of the instrument was studied through a Confirmatory Factor Analysis (CFA), in order to confirm the unidimensional factor structure that had been concluded in the EFA. The estimation method used was ULS. CFI and SRMR were used as fit indices. In addition, due to the importance of studying the factor structure of a construct across different populations, measurement invariance was studied as a function of sex, calculating the configural, metric, and scalar invariance through Multi-Group Confirmatory Factor Analysis (MG-CFA). As these are nested models, a change in CFI of less than − 0.01 (ΔCFI<-0.01) [52] is accepted to assume measurement invariance.

Next, descriptive statistics were used (mean, standard deviation, skewness and kurtosis) for the items. The discrimination indices were analyzed (corrected item-test correlations), being considered adequate when they were over 0.20 [53]. Also, the reliability of the scores was studied through the Cronbach’s alpha coefficient.

Descriptive statistics, discrimination indices, Pearson correlations and reliability were calculated with the SPSS 24 program (IBM Corp, 2016). EFA were calculated with FACTOR program [54]. The CFA and measurement invariance were carried out with the lavaan package in R [55].

## Results

First, both KMO (0.946) and Bartlett’s statistic (*p* < .001) showed a good adequacy of the data to be subjected to Factor Analysis. However, 20 doublets (pair of items) were identified with EREC, showing that their errors are correlated after discounting the common factor (item 27 – item 28; item 24 – item 28; item 14 – item 15; item 2 – item 3; item 25 – item 28; item 20 – item 21; item 12 – item 28; item 24 – item 25; item 15 – item 16; item 10 – item 11; item 26–28; item 5 – item 7; item 7 – item 9; item 26 – item 27; item 6 – item 7; item 23 – item 25; item 3 – item 17; item 1 – item 12; item 5 – item 24; item 20 – item 22). Using a statistical-substantive strategy, 10 items were eliminated on the basis that the content was maintained by the five facets of the scale. Thus, items 28, 25, 15, 20, 7, 3, 10, 26, 12, and 24 were deleted. The EFA was then repeated without these items. All items showed a good fit to the EFA, showing an MSA above 0.90 (Table 1). A single factor explains 55.10% of the total variance, the optimal implementation of the Parallel Analysis recommended a single dimension, and adequate indicators were obtained for the unidimensional structure: UniCo = 0.968, ECV = 0.904, MIREAL = 0.202. Furthermore, the fit of the data to a unidimensional model was satisfactory: CFI = 0.999, and SRMR = 0.048. The factor loadings of the items in the EFA are shown in Table 1. The description of all scales and subscales, along with the reliability coefficients, are summarized in Table 2. All this, together with the high correlations between the five facets of which the READ is composed (Table 3), leads to the conclusion that the scale is essentially unidimensional. Therefore, it seems reasonable to argue for a single dimension.

**Table 1:** Descriptive Statistics, Discrimination Indices, and Factor Loadings of the Items on the READ.

	Original Item	Label	Mean	Standard Deviation	Skewness	Kurtosis	Item-test correlation (corrected)	MSA	Factor loading (EFA)	Factor loading (CFA)
Personal Competence	01	I reach my goals if I work hard	2.16	1.158	1.119	0.537	0.714	0.945	0.78	0.74
	04	I am satisfied with my life up till now	3.06	1.179	0.139	-0.887	0.459	0.935	0.70	0.45
	17	I feel competent	2.51	1.135	0.637	-0.248	0.657	0.929	0.46	0.61
	23	My belief in myself gets me through difficult times	2.65	1.228	0.468	-0.711	0.681	0.948	0.74	0.62
Structure Style	02	I am at my best when I have clear aims and objectives	2.56	1.065	0.550	-0.302	0.673	0.938	0.66	0.80
	08	I always make a plan before I start something new	2.66	1.154	0.513	-0.512	0.674	0.965	0.73	0.73
	13	I am good at organising my time	2.51	1.121	0.690	-0.127	0.692	0.949	0.78	0.68
	18	In my family we have rules that simplify everyday life	2.48	1.159	0.642	-0.343	0.723	0.906	0.49	0.63
Social Support	09	My friends always stick together	2.37	1.103	0.846	0.189	0.730	0.971	0.80	0.66
	14	I have some close friends/family members that really care about me	2.78	1.193	0.291	-0.804	0.643	0.960	0.75	0.74
	19	I always have someone that can help me when I need it	2.60	1.103	0.583	-0.225	0.702	0.936	0.66	0.68
Social Competence	06	I easily make others feel comfortable around me	2.82	1.137	0.390	-0.629	0.657	0.963	0.72	0.73
	11	I easily find new Friends	3.07	1.168	0.093	-0.869	0.472	0.956	0.83	0.74
	16	I am good at talking to new people	2.71	1.143	0.436	-0.604	0.675	0.950	0.73	0.65
	22	I always find something fun to talk about	2.36	1.176	0.773	-0.135	0.761	0.960	0.80	0.77
Family Cohesion	05	In my family we share views of what is important in life	2.63	1.066	0.625	-0.158	0.717	0.956	0.83	0.78
	21	My family view the future as positive, even when very sad things happen	2.31	1.215	0.808	-0.228	0.741	0.940	0.68	0.81
	27	In my family we like to do things together	2.38	1.147	0.721	-0.193	0.739	0.962	0.80	0.79
	Total	Resilience	46.64	14.74	0.724	0.320				
Excluded items										
3		I have some friends/family members that usually encourage me	2.57	1.04	0.61	− 0.06				
7		I know how to reach my goals	2.49	1.10	0.68	− 0.17				
10		I feel comfortable with my family	2.70	1.18	0.46	− 0.64				
12		When it is impossible for me to change certain things I stop worrying about them	2.92	1.15	0.20	− 0.76				
15		In my family we agree on most things	2.68	1.19	0.51	− 0.55				
20		When I have to choose between several options I almost always know what will be right for me	2.27	1.15	0.83	− 0.02				
24		In my family we support each other	2.37	1.15	0.71	− 0.22				
25		I always find something comforting to say to others when they are sad	2.69	1.14	0.35	− 0.59				
26		When things go badly I have a tendency to find something good that can come out of it	2.63	1.19	0.40	− 0.66				
28		I have some close friends/family members that value my qualities	2.50	1.20	0.55	− 0.53				

Second, the unidimensional factor structure of the instrument was tested through a CFA. As reflected in Table 1, the factor loadings are all very high [0.450 − 0.881], the fit being adequate (CFI = 0.852; TLI = 0.832; RMSEA = 0.112 [0.104 − 0.120]; SRMR = 0.058). On the other hand, once the unidimensional factor structure had been verified, we went on to study the measurement invariance as a function of gender. The three levels of invariance studied (configural, metric and scalar) were fulfilled as a function of gender (Table 4).

Thirdly, the descriptive statistics of the original READ-28 items and as well final READ-18 items retained through CFA were studied (Table 1). The discriminative power was very high for each of the items (Item Discrimination (I.D.) [0.459 − 0.761]). As for the reliability of the scores, the Cronbach’s alpha coefficient showed adequate internal consistency of the scores (α = 0.943).

**Table 2:** Description and reliability coefficients of all scales and subscales

Measure	Number of items in the scale	Likert scale value	n	Mean total score ± SD [range]	Cronbach’s alpha
READ – 18 Subscales					
Personal Competence	4	1–5	546	10.38 ± 3.46 [4–20]	0.72
Structure Style	4	1–5	546	10.21 ± 3.54 [4–20]	0.79
Social Support	3	1–5	546	7.75 ± 2.75 [3–15]	0.73
Social Competence	4	1–5	546	10.96 ± 3.50 [4–20]	0.75
Family cohesion	3	1–5	546	7.33 ± 2.90 [3–15]	0.80
Total READ-18	18	1–5	546	46.64 ± 14.73 [18–90]	0.94
Total READ-28	28	1–5	546	72.45 ± 22.81 [28–140]	0.96
DASS-8	8	0–3	546	7.37 ± 5.65 [0–24]	0.87
Depression	3	0–3	546	2.71 ± 2.39 [0–9]	0.76
Anxiety	3	0–3	546	2.48 ± 2.32 [0–9]	0.76
Stress	2	0–3	546	2.18 ± 1.73 [0–6]	0.63
CRIES-13	13	0–5	546	17.19 ± 14.75 [0–65]	0.92

Cronbach’s alpha for total items of READ-18 was 0.94 and 0.96 for READ-28. This finding indicates that despite excluding 10 items, the Cronbach’s alpha for READ-18 showed same high level of consistency across scale’s items

**Table 3:** Pearson Correlations between Facets of READ.

	Structure Style	Social Support	Social Competence	Family Cohesion
Personal Competence	0.820	0.770	0.775	0.797
Structure Style		0.805	0.788	0.807
Social Support			0.784	0.791
Social Competence				0.770

**Table 4:** Measurement Invariance of READ Based on Sex

		CFI	ΔCFI	RMSEA	Δ RMSEA
	Configural	0.839		0.118	
	Metric	0.838	− 0.001	0.115	− 0.003
	Scalar	0.839	0.001	0.111	− 0.004

### Concurrent validity

Higher resilience scores (lower resilience) were significantly associated with more stress (*r* = .09; *p* = .033), anxiety (*r* = .22; *p* < .001), depression (*r* = .13; *p* = .003), total psychological distress (DASS-8 total score) (*r* = .17; *p* < .001) and CRIES-13 scores (*r* = .11; *p* = .009).

## Discussion

The primary objective of the present study was to assess the psychometric properties of the Arabic version of the READ among a group of non-clinical Arabic-speaking Lebanese adolescents aged 12–18 years. The results indicated that the scale presented excellent reliability and provided evidence supporting its factorial and convergent validity as well as measurement invariance across gender groups. Consequently, the findings strongly suggest that the READ can be utilized as a reliable and valid tool to evaluate Arab adolescents’ individual, family and social resilience resources.

With regard to factorial validity of the Arabic version of the READ, the results showed support to a unidimensional factor structure. Upon removing 10 items from the scale while retaining the five facets of the scale, the exploratory factor analysis (EFA) showed a better fit compared to the 28-item scale with the five facets showing strong correlations among themselves. This is quite similar to the results of Moksnes and Haugan (2017) who found that, upon removing 8 items from the scale, a better model fit resulted compared to the 28-item model [16]. Similarly, another study validating the READ found that the exclusion of 5 items resulted in the best model fit for the scale [30]. The elimination of the 10 items is the result of having studied the doublets of items through the EREC method. These items present a substantive overlap and their elimination should not be problematic and thus the reduction of the scale. The READ scale still covers all substantive aspects with 18 items (Personal Competence, Structure Style, Social Support, and Social Competence). Our modified 18-item unidimensional factor structure of the scale was then tested through confirmatory factor analysis (CFA) which resulted in very high factor loadings. More specifically, by looking closer at the factor loadings, it is clear that, in both samples, fair to good values ensued with all of the 18 items in the modified scale, with 13 items having a factor loading higher than 0.7 in the first sample, and 10 items in the second sample. This unidimensional model points out that the scale should be regarded as measuring one, unique aspect of resilience. However, further examination and evaluation of the adjusted and revised factor structure of the scale is crucial, particularly with regards to assessing the instrument’s validity and stability in diverse samples of adolescents. Additionally, our findings highlight that the unidimensional factor structure of the Arabic version of the READ was identical across male and female adolescents at the three levels of invariance (Configural, Metric and Scalar). There were no significant gender differences in READ scores, confirming that, for boys and girls, the factor structures were similar. This is concordant with the results of other studies that also showed that the READ is invariant across genders [30, 31].

As for the reliability of the Arabic version of the READ, strong internal consistency of the scores was measured with a Cronbach’s alpha of 0.943. This is similar to the findings of other studies that showed adequate reliability of the READ scale [16, 31, 32]. The DASS-8 and CRIES-13 were utilized for the calculation of the convergent validity of the Arabic version of the READ. As anticipated, the results of this study suggest that higher levels of resilience were associated with lower levels of psychopathology, namely depression, anxiety, stress and PTSD symptoms. Comparably, other studies have found a similar, inverse association between resilience and various negative (psychopathology) indicators, suggesting that resilience acted as a protective factor against symptoms of psychopathology [30, 33, 56]. In light of adverse life events, resilience is regarded as a safeguarding element that fosters positive results that enable individuals to healthily cope with the challenging life experiences [57], shielding them from the emergence of trauma-related psychological disorders [58]. This is in line with the definition of resilience as a concept that represents one’s personal capacity to deal with and overcome adverse life circumstances [59]. These results, and the availability of a significant association between the Arabic version of the READ with other psychopathological instruments supports the validity of the READ as a measurement of Arab adolescents’ personal and social resilience resources, allowing for its use in an Arabic framework.

### Study limitations

The present study has some limitations that could be improved upon in future research. Considering the recruitment method used to gather participants, it can be assumed that the sample does not represent the general Lebanese population or adolescents. Additionally, even if this study identified a good model fit based on the gathered data and scores, it does not mean that this is the best and only model for the Arabic version of the READ scale. Therefore, future research might be able to identify another good model fit for the scale. Additionally, considering that the questionnaire consisted of self-reported scales, there is a likelihood that the participants fell for the self-reporting bias [16]. Depression, anxiety and stress were evaluated using 2–3 items. Adding to that, it is recommended in future studies to assess the clinical profile of the participants (if they self-report any psychopathology) and the adolescents’ exposure to adversities (such as abuse, neglect, chronic stress, etc.). In fact, screening children for exposure to adversity provides education for parents and caregivers about the relationship between early adversity and negative health outcomes. Second, it informs the healthcare practitioner’s risk assessment and plan of care by identifying patients who are at high risk for a toxic stress physiology, which may be an underlying root cause of clinical symptomatology [60]. Finally, although a nonclinical sample of individuals exhibits a common resilient trait characterized by positive self-perception and optimism towards the future, future studies might need to focus on recruiting a sample of adolescents that has experienced a significant amount of stress and managed to cope with it [34].

## Conclusion

The current study’s findings provide evidence of the psychometric validity of the Arabic version of the READ, supporting its suitability for evaluating adolescents’ personal and social resilience resources based on their experiences over the past month. We hope that the availability of the Arabic version of the READ will facilitate the comprehensive exploration of correlations and relationships between resilience and a variety of other psychopathologies and sociodemographic variables within a cultural and linguistic context, and will encourage and promote international comparisons and research collaborations involving Arab countries.

## Data Availability

The datasets generated and/or analysed during the current study are not publicly available due to restrictions from the ethics committee but are available from the corresponding author on reasonable request.
